# Electrocardiographic Markers of Sudden Unexpected Death Risk in Pediatric Epilepsy: A Comparative Study of Generalized and Focal Seizures

**DOI:** 10.3390/diagnostics15131622

**Published:** 2025-06-26

**Authors:** Serra Karaca, Doruk Özbingöl, Pelin Karaca Özer, Mustafa Lütfi Yavuz, Kemal Nişli, Kazım Öztarhan, Çisem Duman Kayar, Ceyda Öney, Edibe Pempegül Yıldız

**Affiliations:** 1 Department of Pediatric Cardiology, Istanbul Faculty of Medicine, Istanbul University, 34093 Istanbul, Turkey; kemalnisli@yahoo.com (K.N.); oztarhank@gmail.com (K.Ö.); 2 Department of Cardiology, Istanbul Faculty of Medicine, Istanbul University, 34093 Istanbul, Turkey; pkaracaozer@gmail.com (P.K.Ö.); mustafayavuz135@gmail.com (M.L.Y.); 3 Department of Pediatric Neurology, Istanbul Faculty of Medicine, Istanbul University, 34093 Istanbul, Turkey; cisemduman@gmail.com (Ç.D.K.); ceydaoney@hotmail.com (C.Ö.); edibepembegul@hotmail.com (E.P.Y.)

**Keywords:** electrocardiography, epilepsy, sudden death, antiepileptic drugs

## Abstract

**Background/Objectives**: Sudden unexpected death in epilepsy (SUDEP) is a major cause of mortality in pediatric epilepsy. Cardiac arrhythmias, possibly reflected by electrocardiographic (ECG) abnormalities, are thought to contribute significantly to SUDEP risk. This study aimed to evaluate ECG indices associated with an increased risk of both atrial and ventricular arrhythmias and sudden cardiac death in pediatric patients with generalized and focal seizures, excluding those with underlying channelopathies. **Materials and Methods**: Pediatric patients aged 0–18 years with generalized or focal epilepsy followed at our center between October 2024 and April 2025 were enrolled. Comprehensive cardiac evaluations, including echocardiography and 12-lead ECG, were conducted. Patients with channelopathies, structural heart defects, or significant congenital heart disease were excluded. ECG parameters—QT dispersion (QT Disp), corrected QT interval (QTc), QTc dispersion (QTc Disp), P-wave dispersion (P Disp), and T peak-T end interval (Tp-e)—were analyzed across epilepsy subgroups and compared to healthy controls. Effects of antiepileptic drug (AED) use and gender were also assessed. **Results**: A total of 151 participants were included (generalized: *n* = 51; focal: *n* = 50; controls: *n* = 50). QTc and Tp-e intervals were prolonged in both epilepsy groups compared to controls (*p* = 0.001 and *p* = 0.036, respectively), however, they fell within the conventional parameters. AED use was associated with further prolongation of QTc (*p* = 0.035) and Tp-e (*p* = 0.037), these metrics were similarly found to be within the established normative boundaries. Phenobarbital and lamotrigine users showed the longest QTc, albeit not statistically significant. Males with generalized seizures had longer maximum P-wave duration (P Max) than females (*p* = 0.009). A moderate correlation was found between Tp-e and QTc (r = 0.557, *p* = 0.001). **Conclusions**: Although there are findings in our study that may suggest a relationship between SUDEP and arrhythmia according to electrocardiographic markers associated with arrhythmia risk, larger and prospective studies with long-term follow-up are needed in the future.

## 1. Introduction

Sudden unexpected death in epilepsy (SUDEP) is recognized as one of the most devastating outcomes in patients with epilepsy, particularly among children and young adults. Although the precise mechanisms underlying SUDEP remain incompletely understood, increasing evidence suggests that cardiac arrhythmias may play a significant role, particularly through electrocardiographic (ECG) abnormalities that predispose to fatal arrhythmias [1,2]. Several studies have indicated that parameters such as QT interval dispersion (QT Disp), P-wave dispersion (P Disp), and Tpeak–Tend interval (Tp-e) may serve as noninvasive markers of heightened arrhythmic risk in epileptic populations [3,4,5].

Importantly, epilepsy itself may exert deleterious effects on autonomic cardiac regulation, independent of structural heart disease or channelopathies. Both generalized and focal epilepsies have been implicated in autonomic dysfunction, although the extent and pattern of cardiac involvement may differ between epilepsy types [6,7]. Moreover, interictal ECG abnormalities have been reported even in patients without overt channelopathy or structural heart defects, suggesting intrinsic epileptic mechanisms affecting cardiac electrophysiology [8].

Generalized seizures are defined as originating at some point within, and rapidly engaging, bilaterally distributed networks, which can include cortical and subcortical structures, but not the entire cortex. Focal seizures are defined as originating within net-works limited to one hemisphere [9].

Previous research has largely focused on generalized epilepsy, particularly tonic–clonic seizures, in relation to SUDEP risk. Research has indicated that experiencing three or more generalized tonic–clonic seizures (GTCS) annually appears to constitute the most significant weighted risk factor for SUDEP, with the subsequent risk being associated with having ≥13 seizures of any type within the preceding 12 months. Furthermore, the phenomenon of polypharmacy is recognized as a critical risk factor, with evidence demonstrating that the risk of SUDEP is heightened in individuals who are prescribed three or more antiepileptic medications in contrast to those on monotherapy. Additionally, developmental delay has been posited as a potential risk factor. Children diagnosed with complex epilepsy, particularly those concomitantly affected by neurodisability, may exhibit an elevated risk for SUDEP [10].

Inventories are currently being developed to assist in the identification of individuals who are at risk. DeGiorgio et al. and Novak et al. were the first to document the SUDEP inventory [11,12]. The most significant weighted risk factors consist of epilepsy duration exceeding 50 seizures of any classification per month over the prior 12 months, alongside experiencing more than three tonic–clonic seizures within the last year. The SUDEP-7 inventory has demonstrated a correlation with biomarkers indicative of SUDEP risk, notably the root mean square of successive RR interval differences (RMSSD), which serves as a metric for vagus-mediated heart rate variability and is significantly diminished in cases of drug-resistant epilepsy. Additionally, the SUDEP-7 inventory is linked to postictal generalized suppression, which is another biomarker associated with sudden death. Although the SUDEP-7 inventory necessitates external validation within independent cohorts, recent findings suggest a strong correlation between the completion of the inventory by physicians and patients [11,12,13,14].

In investigations concentrating on the pathophysiology and electroclinical risk determinants for SUDEP, alongside cardiac biomarkers, postictal generalized electroencephalographic suppression (PGES) has recently gained significant attention. The conceptualization of PGES was initially articulated by Lhatoo et al., defining it as “the postictal generalized absence of electroencephalographic activity <10 μV in amplitude, immediately or within 30 s subsequent to the cessation of an ictal EEG pattern.” Empirical research has demonstrated that PGES is commonly detected in individuals diagnosed with generalized convulsive seizures (GCS). Upon comprehensive examination of all seizures, the incidence of PGES exceeding 80 s resulted in a fourfold increase in the adjusted odds of SUDEP when contrasted with PGES surpassing 50 s. Extended duration of the tonic phase, age at which epilepsy commences, presence of postictal immobility, and episodes of oxygen desaturation are correlated with the incidence of PGES in patients experiencing GCS [15]. PGES has been established as a prevalent, albeit variable, phenomenon subsequent to convulsive seizures. Nevertheless, the correlation between particular seizure attributes, cardiorespiratory impairments, autonomic dysregulation, the administration of antiepileptic drugs (AEDs), and PGES continues to be ambiguous, with existing findings being scant and predominantly incongruous. The heterogeneity observed in the presence of PGES subsequent to a seizure, the absence of methodical documentation of physiological parameters both during and in the intervals between seizures, and the incorporation of inadequately defined patient cohorts represent the principal constraints of the evidence amassed to date [16].

The computation of descriptive indices of heart rate variability (HRV) for children diagnosed with focal seizures in contrast to those with generalized seizures facilitated a distinction between the two forms of epilepsy concerning the autonomic nervous system’s regulation of heart rate during pre-ictal and post-ictal temporal intervals. Recent studies have demonstrated that when compared with patients experiencing focal seizures during the pre-seizure phases, children exhibiting generalized seizures presented with a significantly reduced mean RR interval. This finding indicates that the pre-ictal phases are predominantly characterized for children with generalized epileptic seizures by a pronounced shift in the sympatho-vagal equilibrium towards sympathetic predominance and a withdrawal of vagal tone. The identification of these discrepancies in temporal windows distanced from the seizure onset may suggest that in cases of generalized epilepsy, tachycardia and sympathetic hyperactivity are prevalent features of inter-ictal periods. Consequently, it appears that the engagement of extensive neural structures in the context of epilepsy exerts a more substantial influence on the heart rate fluctuations associated with seizures, as corroborated by earlier investigations that reported significantly elevated ictal tachycardia in generalized seizures when compared to non-generalized seizures [17].

Data directly comparing ECG-derived arrhythmogenic indices between generalized and focal epilepsy in pediatric populations remain scarce. Understanding whether specific ECG markers differ between these groups could enhance risk stratification and inform targeted surveillance strategies. In this study, we aimed to systematically compare QT Disp, corrected QT interval (QTc), QTc dispersion (QTc Disp), Tp-e, and P Disp between pediatric patients diagnosed with generalized or focal epilepsy who do not have underlying channelopathies.

By elucidating potential ECG differences between epilepsy subtypes, we hope to contribute to the early identification of children at greater risk for arrhythmic events and SUDEP. To our knowledge, this is the first study in the literature to compare ECG data of pediatric patients with generalized and focal epilepsy who do not exhibit channelopathies in terms of sudden death risk and to also evaluate factors such as medication use, monitoring period, and gender.

## 2. Material and Methods

Patients aged 0 to 18 years who were followed up with a clinical diagnosis of epilepsy at our pediatric neurology outpatient clinic from October 2024 to April 2025 constituted the population of our research. Each participant underwent comprehensive evaluation via echocardiography and electrocardiography conducted in our pediatric cardiology clinic. The patients diagnosed with epilepsy were categorized into two distinct groups based on seizure type: generalized and focal seizures.

Individuals diagnosed with channelopathy were systematically excluded from the study. Exclusion of channelopathies was done by genetic screening.

The criteria for exclusion also encompassed abnormal laboratory findings that could potentially influence the ECG, including but not limited to abnormalities in calcium, potassium, magnesium, and blood glucose levels, as well as a documented history of pharmacological interventions involving anti-psychotic medications, anti-arrhythmic agents, and antibiotics such as aminoglycosides, in addition to anti-depressants. Furthermore, individuals with traumatic injuries, meningitis, encephalitis, seizure-related syndromes, and structural anomalies were excluded from the study. In addition, participants with hemodynamically significant congenital heart disease and cardiomyopathy were also excluded. Only those with normal echocardiographic findings were included in the analysis.

A comparative assessment was conducted between the patients exhibiting generalized and focal seizures and a third cohort comprising healthy children matched by age. Patients underwent evaluation utilizing ECG data, which was analyzed in relation to the duration of follow-up and the administration of antiepileptic pharmacotherapy, alongside the categorization of seizure types. Specifically, patients who had been seizure-free for at least one year and showed no epileptiform activity on electroencephalogram (EEG) were monitored without antiepileptic medication. In contrast, those who continued to experience seizures were treated with antiepileptic drugs (AED). It is noteworthy that no classification was implemented based on the frequency of seizure occurrences.

All participants underwent a standardized routine 12-lead body surface electrocardiogram (ECG) Kenz Cardico 1215 recorded at a paper speed of 50 mm/s. Three leads were recorded concurrently. The duration of the P-wave, the PR interval, the duration of the QRS complex, and the QT interval were computed and subsequently adjusted for heart rate variations. The measurements were done during the preictal stage. One investigator (PKÖ) who was blinded to the clinical status of the patients, manually measured the durations of the P- and QT-waves. To enhance measurement accuracy, calipers and a magnifying lens were utilized to delineate the ECG deflections. The initiation of the P-wave was defined as the point of intersection between the isoelectric line and the commencement of P-wave deflection, while the termination of the P-wave was demarcated as the point of intersection between the conclusion of the P-wave deflection and the isoelectric line. The maximum P-wave duration (P Max) from any of the 12-lead surface ECGs was computed and employed as an indicator of prolonged atrial conduction time. The variable P disp, which is defined as the differential between P Max and minimum P-wave duration (P Min), was calculated from the 12-lead ECG. All QT interval measurements were conducted manually utilizing calipers. The average value of the three QT intervals was determined for each electrocardiographic lead. The QT interval was assessed from the onset of the QRS complex to the conclusion of the T wave and was corrected for heart rate employing Bazett’s formula. QT Disp was characterized as the maximal inter-lead disparity in QT intervals. QTc values were calculated using Bazett’s formula (corrected QT [QTc] = QT/√(R − R) interval).

The Tp-e interval (milliseconds) was determined utilizing the tangent method. The duration from the apex of the T wave (or the nadir if a negative or biphasic T wave was recorded) to the point of intersection between the tangent at the steepest segment of the T wave and the isoelectric line was digitally quantified in milliseconds employing Cardio Calipers Version 3.3 software (Iconico, Inc., New York, NY, USA). The Tp–e interval was assessed based on the most optimal T wave observed in lead DII. In circumstances where lead DII was deemed unsuitable for analysis, precordial lead V5 was employed. Measurement of the Tp-e interval was excluded for leads exhibiting T waves with amplitudes below 1.5 mm. The 12-lead ECG parameters were evaluated by an independent observer (PKÖ) who remained blinded to the clinical data. The recorded Tp-e value represented the highest measurement acquired through two assessments conducted by the observer.

Statistical analyses were performed using IBM SPSS Statistics (Version 27.0, IBM Corp., Armonk, NY, USA). The normality of continuous variables was assessed using the Shapiro–Wilk test. Variables with normal distribution were presented as mean ± standard deviation and analyzed using parametric tests (independent-samples *t*-test or one-way ANOVA). Variables that did not meet the assumption of normality were expressed as median (minimum–maximum) and compared using non-parametric tests (Mann–Whitney U test or Kruskal–Wallis test).

For comparisons involving more than two groups, post hoc pairwise analyses were conducted following the Kruskal–Wallis test. Categorical variables were summarized as frequencies and percentages, and compared using the chi-square test.

Age-based subgroup analysis was performed by categorizing participants into three groups: Group 1 (0–5 years), Group 2 (6–12 years), and Group 3 (13–18 years), and ECG parameters were compared accordingly.

Correlation analyses between continuous variables, including antiepileptic drug use duration and electrocardiographic parameters, were carried out using Spearman’s rank correlation coefficient due to the non-normal distribution of most variables. The strength of the correlations was interpreted as follows: coefficients < 0.30 were considered weak, 0.30–0.59 moderate, and ≥0.60 strong. A two-tailed *p*-value < 0.05 was considered statistically significant throughout the analysis.

## 3. Results

Table 1 summarizes the comparison of demographic and electrocardiographic parameters among control subjects and patients with generalized or focal seizures. There were no statistically significant differences between the groups in terms of age, sex distribution, weight, or height (*p* = 0.71; *p* = 0.59; *p* = 0.29; *p* = 0.41, respectively).

Although QTc intervals were within normal limits in all groups, it was longest in the focal seizure group and shortest in the control group, and was significantly different among the three groups (*p* < 0.001).

The Tp-e interval also showed statistically significant differences among groups (*p* = 0.036). The Tp-e was significantly longer in generalized and focal seizure groups compared to the controls.

No statistically significant differences were found in QT Disp, QTc Disp, P Disp, or P Max values across the groups. However, a borderline difference was noted for P Disp (*p* = 0.053).

A statistically significant difference was found in P Min (*p* = 0.01), with lower values observed in the control group and focal seizure group when compared to the generalized seizure group.

The duration of follow-up did not differ significantly between the seizure groups (*p* = 0.07).

Table 2 presents the comparison of demographic, clinical, and electrocardiographic characteristics among patients using different AEDs. A statistically significant difference was found in age among the AED groups (*p* = 0.04), with patients receiving clonazepam and lamotrigine being older on average compared to those receiving other medications. However, no statistically significant differences were found in weight (*p* = 0.23) or height (*p* = 0.42) across the treatment groups.

Although there were numerical differences in QTc values, these did not reach statistical significance (*p* = 0.24). The highest QTc values were observed in patients on phenobarbital and clobazam.

Tp-e intervals were comparable across all AED groups, with no significant difference (*p* = 0.59). Similarly, there were no statistically significant differences in QT Disp, QTc Disp, or P Disp (*p* = 0.89; *p* = 0.74; *p* = 0.99, respectively).

P Min and P Max durations also did not differ significantly among the AED groups (*p* = 0.75; *p* = 0.85, respectively).

Monitoring period durations varied, with the longest follow-up observed in patients using clonazepam, though the difference was not statistically significant (*p* = 0.20).

Table 3 presents the correlation analysis between the duration of AED use and various ECG parameters. No statistically significant correlations were found between the monitoring period and any of the evaluated ECG parameters.

Specifically, the duration of AED use showed no significant correlation with QTc (r = –0.011, *p* = 0.92), Tp–e interval (r = –0.060, *p* = 0.55), QT Disp (r = –0.040, *p* = 0.69), or QTc Disp (r = –0.133, *p* = 0.19). Similarly, no significant correlations were observed between the duration of drug use and P Disp (r = –0.059, *p* = 0.56), P Min (r = 0.060, *p* = 0.55), P Max (r = –0.014, *p* = 0.89).

Table 4 compares control subjects, patients not using antiepileptic drugs (No-AED), and patients receiving AED in terms of demographic and electrocardiographic parameters.

There were no statistically significant differences among the three groups in terms of age, weight, or height (*p* = 0.72; *p* = 0.42; *p* = 0.47, respectively).

However, a statistically significant difference was observed in the QTc interval (*p* = 0.035). Both the No-AED and AED groups had significantly higher QTc values compared to the control group, with the highest values seen in the AED group. A similar pattern was observed for the Tp–e interval, which differed significantly across groups (*p* = 0.037). The Tp–e interval was significantly prolonged in both the No-AED and AED groups compared to the control group. Despite the statistical significance, both parameters remained within clinically acceptable ranges.

There were no significant differences in monitoring period, QT Disp, QTc Disp, P Disp, P Min, or P Max duration (*p* = 0.13; *p* = 0.45; *p* = 0.09; *p* = 0.25; *p* = 0.16; *p* = 0.77).

The following electrocardiographic measurements were evaluated according to sex in both focal and generalized seizure groups: QTc interval, Tp-e interval, monitoring duration, QT and QTc dispersions, and P-wave parameters (Table 5).

In the focal seizure group, female patients had a median QTc interval of 410 ms (384–440), compared to 402 ms (380–440) in males (*p* = 0.24). The Tp-e interval was slightly higher in females (64.9 ± 2.7 ms) than in males (63.9 ± 2.4 ms, *p* = 0.19). The median duration of follow-up was also longer among females [6 years (0.5–17) vs. 3 years (0.5–13), *p* = 0.06]. No statistically significant sex differences were observed in QT Disp (29.8 ± 3.4 vs. 31.6 ± 4.7, *p* = 0.15), QTc Disp [36 ms (27–45) vs. 38 ms (28–48), *p* = 0.67], P Disp [31 ms (23–38) vs. 32 ms (24–41), *p* = 0.68], P min [55 ms (48–61) vs. 55 ms (47–59), *p* = 0.93], or P max [86 ms (78–90) vs. 86 ms (80–90), *p* = 0.84].

In the generalized seizure group, QTc values were similar between females [398 ms (370–451)] and males [390 ms (380–435), *p* = 0.96], as were Tp-e intervals (64.3 ± 3.3 ms vs. 64.4 ± 3.2 ms, *p* = 0.93) and follow-up duration [4 years (1–10) vs. 3 years (1–7), *p* = 0.19]. QT and QTc Disp, as well as P Disp and P Min, did not differ significantly between sexes. However, P Max duration was significantly longer in males [87 ms (82–92)] than in females [84 ms (78–91), *p* = 0.009].

The correlation between Tp–e interval and other ECG parameters was analyzed (Table 6).

A moderate positive correlation was found between Tp–e and QTc interval (r = 0.557, *p* < 0.001), indicating that longer QTc intervals are associated with prolonged Tp–e intervals. Additionally, Tp–e showed a statistically significant but weaker positive correlation with QTc Disp (r = 0.269, *p* < 0.001).

In contrast, no significant correlations were observed between Tp–e and QT Disp (r = 0.092, *p* = 0.26), P Disp (r = –0.054, *p* = 0.51), P Min (r = 0.042, *p* = 0.61), or P Max (r = –0.023, *p* = 0.78).

Table 7 presents the distribution of ECG parameters across three age-based subgroups: Group 1 (0–5 years), Group 2 (6–12 years), and Group 3 (13–18 years). No statistically significant differences were observed among the age groups in terms of QTc (*p* = 0.40) or Tp–e interval (*p* = 0.38), although numerically higher QTc and Tp–e values were noted in the youngest age group (0–5 years). Similarly, there were no significant differences in QT Disp (*p* = 0.98), QTc Disp (*p* = 0.78), P Disp (*p* = 0.54), P Min (*p* = 0.29), or P Max (*p* = 0.83) among the three age subgroups. These results indicate that ECG repolarization and atrial conduction parameters remain relatively stable across pediatric age groups in this cohort, suggesting age does not significantly affect these ECG markers.

## 4. Discussion

This investigation undertook a comprehensive analysis of an extensive dataset of ECG in order to assess the likelihood of arrhythmogenic events in pediatric patients diagnosed with epilepsy. The paramount findings derived from our research may be encapsulated in the following manner:

1. The QTc interval exhibited a notable prolongation in individuals experiencing generalized and focal seizures when contrasted with the control group. Nevertheless, despite achieving statistical significance, the results were determined to fall within the parameters of normalcy. Also, the Tp-e interval was markedly extended in both generalized and focal seizure groups in comparison to the controls.
2. The QTc interval and Tp-e interval exhibited a statistically significant prolongation within the AED cohort in comparison to the control cohort, but they were found to be within normal limits.
3. In the comparative analysis of various antiepileptic medications, the QTc interval was observed to be the most prolonged in subjects administered phenobarbital and lamotrigine; however, the disparities among the cohorts did not reach statistical significance. In a similar vein, the Tp-e intervals were found to be analogous.
4. P Max exhibited a statistically significant elevation in males in comparison to females in the generalized seizure cohort.
5. Correlation analysis revealed a robust positive relationship between the Tp-e interval and the QTc interval, in addition to a comparatively weaker yet statistically significant correlation with QTc Disp.

The conceptualization of SUDEP has been delineated through specific criteria established within the academic literature [18]. Criteria:

1. The victim had epilepsy, defined as recurrent unprovoked seizures.
2. The victim died unexpectedly while in a reasonable state of health.
3. The death occurred “suddenly” (in minutes), when known.
4. The death occurred during normal activities (e.g., in or around bed, at home, at work) and benign circumstances.
5. An obvious medical cause of death was not found.
6. The death was not directly caused by the seizure or status epilepticus. Definite SUDEP meets all above criteria, with post-mortem examination.

A comparative summary of electrophysiological findings, cardiac autonomic dysfunction, and associated SUDEP risk across different epilepsy types is presented in Table 8.

Epilepsy constitutes a prevalent neurological disorder among pediatric populations globally and represents one of the most significant and intricate medical conditions that can, at times, culminate in unexpected mortality [25].

The volume of evidence elucidating the significance of ion-channel abnormalities in the etiology of epileptic disorders is experiencing a rapid escalation [26]. Recent investigations in the field of genetics have elucidated that in the majority of individuals afflicted with epilepsy, the genetic loci encoding various ion channel proteins exhibit alterations. For instance, the sodium channel 1 subgroup (*SCN1B*) is located on the 19th chromosome, while the 1 subgroup (*SCN1A*) resides on the 2nd chromosome; both have been identified as defective in cases of generalized epilepsy associated with febrile convulsions. Furthermore, the genes responsible for encoding potassium channels, specifically *KCNQ2* and *KCNQ3*, are situated on the 20th and 8th chromosomes, respectively, and these genes are found to be impaired in patients diagnosed with benign neonatal familial convulsions [27]. Certain classifications of ion-channel anomalies are implicated in the pathogenesis of arrhythmias in individuals diagnosed with long QT syndromes [26,28,29,30,31,32,33,34,35]. A minimum of seven distinct long QT syndromes have been characterized, wherein the functionality of potassium and sodium channels is compromised. Pediatric patients exhibiting long QT syndromes may manifest with epileptic seizures, or the onset of epilepsy may be delayed until later developmental stages. Nonetheless, it remains ambiguous whether this phenomenon is a direct consequence of recurrent episodes of ventricular arrhythmia or if there exists a shared etiological factor that precipitates both arrhythmia and epileptic seizures [36]. In the case of Brugada syndrome, mutations affecting sodium and calcium channels are known to induce arrhythmia [37].

Although numerous genetic investigations elucidate the association between channelopathies and epilepsy, there exists a subset of epileptic patients in whom channelopathies remain undetected, and this particular cohort warrants thorough examination to clarify the correlation between epilepsy and sudden death [38].

A recognized correlation exists between epilepsy and arrhythmia. The likelihood of experiencing sudden death among individuals afflicted with epilepsy is determined to be 24 times higher than that of the general population. One of the predominant etiologies of sudden death in pediatric patients with epilepsy is identified as cardiac dysrhythmia [39].

Several risk factors have been delineated in relation to SUDEP: a younger demographic, male gender, the premature manifestation of seizures, the occurrence of generalized tonic–clonic seizures, and a state of being bedridden [10,40]. Nevertheless, these risk factors remain contentious within the framework of pediatric investigations, wherein the specific epilepsy syndrome appears to serve as the most consistent predictor. The elevated risk of SUDEP within the pediatric cohort is particularly pronounced among infants exhibiting generalized tonic–clonic seizures associated with Dravet syndrome and adolescents diagnosed with idiopathic generalized epilepsy [40].

It is widely acknowledged that an elevation in QT interval and P-wave dispersion may serve as indicators of autonomic dysfunction, and that these two phenomena can predispose individuals to the risk of atrial and ventricular dysrhythmias [29,31,41]. Numerous studies have demonstrated that an elevation in QT Disp serves as a robust prognostic indicator for mortality due to cardiac events [42,43]. Bruyne et al., in a longitudinal investigation involving 5523 elderly participants, determined that individuals whose QTc Disp fell within the upper tertile (QT Disp > 59.6 ms) exhibited a relative risk of cardiac mortality of 2.1 in comparison to those within the lower tertile (QT Disp < 39.0 ms) [44]. In another study conducted by Macfarlane et al. in ostensibly healthy cohorts, a QT dispersion exceeding 50 milliseconds is deemed atypical, suggesting an elevated propensity for arrhythmogenic events and unexpected mortality [45]. It is stated in the literature that, the amplification of the QT Disp correlates positively with an increased likelihood of reentry arrhythmias, a phenomenon that exhibits independence from both age and gender [45,46]. In the present investigation, QT Disp and QTc Disp exhibited marginal elevations within the seizure cohorts when juxtaposed with the control group; however, these variations did not attain statistical significance and were below 39.0 ms.

The duration of the P-wave is indicative of the contraction and depolarization processes occurring within the atrial musculature. P Disp signifies the uniformity present in both inter- and intra-atrial conduction pathways [47]. Souza et al. identified a notable extension of the P-wave duration in individuals diagnosed with epilepsy when compared to the control group [48]. In the present investigation, the P Min exhibited a statistically significant increase in the focal seizure cohort in comparison to the control cohort, whereas the P Max remained comparable across all groups. Additionally, this investigation elucidated that the P Max demonstrated a statistically significant increase in males relative to females within the generalized seizure cohort.

In our study, the Tpeak–Tend interval exhibited a statistically significant prolongation in both groups exhibiting seizures when juxtaposed with the control cohort. Tpeak–Tend constitutes a pivotal metric for the assessment of transmural dispersion of ventricular repolarization [47,49]. The primary mechanism underlying the prolongation of the Tp-e interval and the associated ventricular repolarization abnormalities is attributed to the dysregulation of ion transport processes within the cellular structures of distinct layers of the ventricular myocardium [50]. The prolongation of the Tp-e interval was correlated with an elevated burden of premature ventricular contractions (PVCs) in individuals possessing structurally normal cardiac anatomy, and the Tp-e interval has been demonstrated to be a valuable prognostic indicator for the occurrence of sustained idiopathic ventricular tachycardia (VT) in this population [51,52].

AEDs have the potential to induce the emergence of ventricular tachyarrhythmias through their interaction with ionic channels. Furthermore, the concurrent administration of AEDs may elicit a proarrhythmic effect [53]. In the context of our investigation into AED usage, the QTc interval exhibited the greatest duration in patients administered phenobarbital (422.5 ± 4 ms) and lamotrigine (410.3 ± 35 ms); however, the disparities observed among the groups did not reach statistical significance. Although the cohort receiving lamotrigine and clonazepam was of an advanced age compared to the other AED groups, the remaining electrocardiographic parameters were statistically comparable across all AED groups. Lamberts et al. ascertained that the majority of epileptic patients exhibiting significantly prolonged QTc intervals were administered alternative antiepileptic pharmacotherapies rather than those known to prolong the QT interval, and they subsequently postulated that the severity of the epilepsy itself, as opposed to the pharmacological treatments, might be responsible for the observed adverse cardiac effects [54]. The results of our study can also be considered as data supporting this hypothesis. In our study, lamotrigine and phenobarbital were associated with the greatest QTc prolongation among the antiepileptic drugs evaluated. Considering the potential proarrhythmic implications of QTc prolongation, these agents should be used with caution in susceptible patients. In contrast, levetiracetam—shown in previous studies to have minimal cardiac electrophysiological effects—may represent a safer alternative in terms of arrhythmogenic risk. Levetiracetam is a broad-spectrum AED that has gained popularity due to its favorable pharmacokinetic profile and relatively low risk of severe adverse effects. It is effective for both focal and generalized seizures and is often used as a first-line treatment in children. Studies have shown that levetiracetam is associated with a lower risk of SUDEP compared to older AEDs, likely due to its ability to reduce seizure frequency and minimize neurobehavioral side effects. Levetiracetam has been identified as one of the safest antiseizure medications with respect to cardiac electrophysiology, showing no significant effect on QT interval, QT dispersion, or other repolarization markers [3,55].

Neurodevelopmental disorders (NDDs), such as autism spectrum disorder, attention deficit hyperactivity disorder (ADHD), and epilepsy, often co-occur with cardiac autonomic dysfunction, which can increase the risk of SUDEP. Medications used to manage these conditions, particularly those that prolong the QT interval, have been implicated in increasing the risk of life-threatening cardiac arrhythmias. This response explores the implications of such medications, focusing on their impact on SUDEP risk and the mechanisms underlying these adverse effects [56].

The recent SUDEP workshop by the Epilepsy Research Institute brought much-needed attention to people who may be at greater risk of sudden death. This includes people with generalized tonic–clonic seizures, young individuals—especially young men—older adults who may receive less aggressive treatment, people with intellectual disabilities, pregnant women, and those with syndromes like Dravet. Individuals from minority or low-income backgrounds and those with underlying cardiac risk are also considered more vulnerable [57]. Despite how serious SUDEP is, it remains an area with limited research, particularly in the pediatric population. Our study aims to contribute to this gap by drawing attention to ECG changes that may help flag high-risk children earlier and support future preventive strategies.

It is important to recognize some of the limitations of our work, even though it shows notable ECG changes in children with epilepsy. The limited sample size in several drug groups was one of the primary obstacles, particularly for patients taking less widely used antiepileptic medications such phenobarbital or clonazepam. As a result, it is challenging to draw firm conclusions about how each medication may affect ECG results. Additionally, we did not evaluate the frequency or intensity of their seizures, as these factors may have an impact on ECG findings. Also, we used 12-lead resting ECGs. Although useful, this approach just provides a quick overview. A more complete picture of cardiac risk might have been obtained with Holter monitors.

Our study revealed prolongation of QTc and Tp-e intervals in both generalized and focal epilepsy groups, particularly among patients on antiepileptic drugs; nevertheless, they remained within the parameters of conventionality. The robust association observed between Tp-e and QTc intervals represents another significant discovery of the investigation. Although QT and P-wave dispersions appeared less clinically significant, sex-based differences—such as higher P Max in males with generalized seizures—may deserve further investigation.

## 5. Conclusions

In our study, when arrhythmogenic ECG parameters were examined in pediatric epilepsy patients, there were results that may suggest the risk of arrhythmia, but conducting larger and prospective follow-up studies in the future will broaden our horizons in this area and help predict and reduce the risk of arrhythmia and SUDEP.

## Figures and Tables

**Table 1:** Comparison of demographic and electrocardiographic parameters among control, generalized seizure, and focal seizure groups.

VARIABLES (*n*)	Control (50)	Generalized (51)	Focal (50)	*p*-Value
Age	11 (1–18)	12 (1–18)	10.5 (0.5–18)	0.71
Male Sex (%)	25, (50%)	21, (42%)	25, (50%)	0.59
Weight	39.5 (11–57)	40 (12.5–100)	40.5 (6–89)	0.29
Height	147.5 (85–166)	151 (82–186)	142 (69–190)	0.41
QTc, (ms)	391.5 (370–432) ^b^	396 (370–451) ^c^	405.5 (380–440) ^bc^	<0.001
Tp-e, (ms)	63 ± 2.9 ^ab^	64.3 ± 3.2 ^a^	64.4 ± 2.6 ^b^	0.036
Monitoring Period (years)	-	4 (1–10)	5 (0.5–17)	0.07
QT Disp	29.8 ± 5	31 ± 6.1	30.7 ± 4.2	0.498
QTc Disp	35 (24–45)	38 (23–45)	36.5 (27–48)	0.08
P Disp	33 (14–42)	32 (22–44)	32 (23–41)	0.053
P Min	54 (48–60) ^b^	54 (47–60)	55 (47–61) ^b^	0.01
P Max	86 (74–92)	86 (78–92)	86 (78–90)	0.68

^a^ Statistical significance between control group and generalized group. ^b^ Statistical significance between control group and focal group. ^c^ Statistical significance between generalized group and focal group.

**Table 2:** Comparison of demographic, clinical, and electrocardiographic characteristics according to antiepileptic drug use.

VARIABLES (*n*)	Valproic Acid (28)	Levetiracetam (40)	Clobazam (5)	Carbamazepine (6)	Lamotrigine (3)	Phenobarbital (2)	Clonazepam (2)	*p*-Value
Age	11 (1–15)	9.5 (0.5–17)	12 (4–16)	14.5 (5–18)	16 (14–18)	9.5 (3–16)	17 (16–18)	0.04
Weight	40.5 (13–80)	38 (6–93)	44 (21–81)	46.5 (18–89)	53 (52–72)	39.7 (14–65.3)	60 (46–74)	0.23
Height	149.5 (82–186)	136.5 (69–190)	146 (110–160)	153 (123–176)	157 (150–161)	126.5 (92–161)	163 (161–165)	0.42
QTc (ms)	393.5 (370–436)	405.5 (375–440)	410 (390–432)	392.5 (380–420)	392 (388–451)	422.5 (420–425)	409 (388–430)	0.24
Tp-e (ms)	64.4 ± 3.1	64.2 ± 3	63.9 ± 3.8	64.1 ± 1.2	66.1 ± 4.4	68.3 ± 1.4	65 ± 3.5	0.59
Monitoring Period (years)	3.5 (1–13)	3 (0.5–13)	5 (2–5)	6.5 (0.5–17)	8 (3–9)	4.5 (3–6)	13 (10–16)	0.20
QT Disp	31.2 ± 6.1	30.9 ± 4.6	28.6 ± 5.1	31 ± 4.1	29 ± 10.8	27.5 ± 6.4	32.5 ± 6.4	0.89
QTc Disp	38 (23–45)	36.5 (28–44)	34 (28–45)	36 (35–48)	36 (27–40)	34.5 (34–35)	40 (36–44)	0.74
P Disp	32 (22–44)	31.5 (23–41)	32 (26–39)	31 (27–38)	32 (28–34)	32 (32–32)	32 (30–34)	0.99
P Min	54.5 (47–61)	55 (47–60)	52 (49–56)	54 (50–56)	55 (49–58)	55.5 (55–56)	53 (52–54)	0.75
P Max	86 (78–92)	86 (78–90)	84 (82–88)	85.5 (80–90)	86 (83–87)	87.5 (87–88)	85 (82–88)	0.85

**Table 3:** Correlation between duration of antiepileptic drug use and electrocardiographic parameters.

VARIABLES	Monitoring Period (Years)	
	R	*p*-Value
QTc (ms)	−0.011	0.92
Tp-e (ms)	−0.060	0.55
QT Disp	−0.040	0.69
QTc Disp	−0.133	0.19
P Disp	−0.059	0.56
P Min	0.060	0.55
P Max	−0.014	0.89

**Table 4:** Comparison of control subjects, patients not using antiepileptic drugs, and patients using antiepileptic drugs in terms of demographic and electrocardiographic parameters.

VARIABLES (*n*)	Control (50)	No-AED (15)	AED (86)	*p*-Value
Age	11 (1–18)	11 (6–17)	11.5 (0.5–18)	0.72
Weight	39.5 (11–57)	44 (21–100)	39.5 (6–93)	0.42
Height	147.5 (85–166)	145 (110–180)	145.5 (69–190)	0.47
QTc (ms)	391.5 (370–432) ^b^	402 (370–440)	400 (370–451) ^b^	0.035
Tp-e (ms)	63.1 ± 2.9 ^b^	64.3 ± 2.3	64.4 ± 3 ^b^	0.037
Monitoring Period (years)	-	5 (2–12)	4 (0.5–17)	0.13
QT Disp	29.8 ± 5.1	31.5 ± 4.8	30.7 ± 5.3	0.45
QTc Disp	35 (24–45)	38 (28–44)	37 (23–48)	0.09
P Disp	33 (14–42)	32 (24–41)	32 (22–44)	0.25
P Min	54 (48–60)	54 (49–57)	54.5 (47–61)	0.16
P Max	86 (74–82)	86 (79–90)	86 (78–92)	0.77

^b^ Statistical significance between control group and AED group.

**Table 5:** Sex-based differences in ECG parameters among patients with focal and generalized seizures.

VARIABLES	Focal Seizures		*p*-Value	Generalized Seizures		*p*-Value
	Female	Male		Female	Male	
QTc (ms)	410 (384–440)	402 (380–440)	0.24	398 (370–451)	390 (380–435)	0.96
Tp-e (ms)	64.9 ± 2.7	63.9 ± 2.4	0.19	64.3 ± 3.3	64.4 ± 3.2	0.93
Monitoring Period (years)	6 (0.5–17)	3 (0.5–13)	0.06	4 (1–10)	3 (1–7)	0.19
QT Disp	29.8 ± 3.4	31.6 ± 4.7	0.15	30.5 ± 5.6	31.7 ± 6.9	0.49
QTc Disp	36 (27–45)	38 (28–48)	0.67	37 (23–44)	39 (25–45)	0.19
P Disp	31 (23–38)	32 (24–41)	0.68	30 (24–44)	33 (22–41)	0.12
P Min	55 (48–61)	55 (47–59)	0.93	53.5 (47–59)	54 (48–60)	0.45
P Max	86 (78–90)	86 (80–90)	0.84	84 (78–91)	87 (82–92)	0.009

**Table 6:** Correlation between Tp-e interval and other electrocardiographic parameters.

VARIABLES	Tp-e (ms)	
	R	*p*-Value
QTc (ms)	0.557	<0.001
QT Disp	0.092	0.26
QTc Disp	0.269	<0.001
P Disp	−0.054	0.51
P Min	0.042	0.61
P Max	−0.023	0.78

**Table 7:** Distribution of ECG parameters across age-based subgroups.

VARIABLES (*n*)	Group 1 (0–5 Years) (21)	Group 2 (6–12 Years) (37)	Group 3 (13–18 Years) (43)	*p*-Value
QTc (ms)	412 (375–436)	400 (370–440)	400 (380–451)	0.4
Tp-e (ms)	65 ± 3.3	64.5 ± 2.8	63.9 ± 2.9	0.38
QT Disp	31 ± 4.5	30.9 ± 4.5	30.8 ± 6.1	0.98
QTc Disp	38 (28–45)	36 (23–45)	38 (25–48)	0.78
P Disp	32 (25–40)	33 (23–44)	32 (22–41)	0.54
P Min	55 (48–60)	54 (47–61)	55 (48–60)	0.29
P Max	86 (78–90)	86 (78–92)	86 (80–90)	0.83

**Table 8:** SUDEP and epilepsy type: Summary table.

Epilepsy Type	Key Findings	Relevant ECG Parameters	Implication for SUDEP Risk	Source
Generalized	Often associated with peri-ictal cardiac abnormalities, such as bradycardia. Refractory generalized epilepsy showed higher QT dispersion and impaired HRV; linked with increased SUDEP-7 risk.	Prolonged QT interval, bradycardia, QTc > 450 ms, QT Disp > 50 ms	Higher risk due to autonomic dysfunction and widespread cortical involvement. QTc prolongation and reduced HRV significantly correlated with SUDEP risk.	Surges et al., 2009 [1]; Devinsky et al., 2016 [19]; Hamdy et al., 2022 [20]
Focal	Can cause localized cortical involvement that affects autonomic regulation. Seizure-induced ictal asystole observed exclusively in focal epilepsies (especially temporal and frontal), often left-lateralized.	Ictal asystole, HR variability, Asystole duration 4–60 s post-seizure	Risk depends on seizure spread; focal seizures may cause bradyarrhythmia or asystole in severe cases. Prolonged postictal periods and central apnea may contribute to reduced cerebral perfusion and SUDEP.	Devinsky et al., 2016 [19]; Dlouhy et al., 2016 [21]; Rocamora et al., 2003 [22]
Both	Alterations in autonomic nervous system function observed in both types. Both ictal and postictal arrhythmias (asystole, AV block, VT/VF) can occur in various types of epilepsy; cardiovascular comorbidities common.	HRV reduction, arrhythmia post-seizure Postictal asystole, QT Disp, VT/VF	Postictal autonomic instability increases SUDEP risk, especially with impaired arousal and cardiorespiratory delay. Postictal EEG suppression and apnea after generalized tonic–clonic seizure linked with terminal asystole; genetic channelopathies may underlie fatal arrhythmias.	Ryvlin et al., 2013 [23]; Shmuely et al., 2017 [24]

## Data Availability

The original contributions presented in this study are included in the article. Further inquiries can be directed to the corresponding author.
